# New York

**Published:** 1898-05

**Authors:** 


### COMMENCEMENT EXERCISES.

---

### NEW YORK COLLEGE OF VETERINARY SURGEONS.

The commencement exercises were held on commencement day, April 1st, in College Hall. Fifteen were eligible for the "'98" examinations. Of this number thirteen presented themselves, eight of whom were successful in obtaining the diploma of the school. Five of the class failed, or 38.5 per cent. Those receiving diplomas were Philip Caspian Finn, Brooklyn, N. Y.; William Lawrence Fowler, Banksville, N. Y.; Niran Odell Gilbert, Irvington-on-Hudson, N. Y.; James Mannington Richardson, Tunbridge Wells, England; Arthur Ward Smith, Bloomfield, N. J.; James Edgar Smith, V. S. (Ontario), Webster, N. Y.; Valentine L. Smith, Baldwins, Long Island, N.Y., and William Henry Wheeler, V.S. (Ontario), Stamford. N.Y.

Dr. James Mannington Richardson won the gold medal for the best general examination. Dr. William Henry Wheeler passed the best practical examination, winning a case of instruments. Mr. Francis C. Edmunds, of Glen Cove, Long Island, N. Y., passed the best second-year examination, receiving the Regents' certificate. Mr. Albert Pfefferkorn, New York City, won the silver medal for the best junior examination.

At 8 P.M. members of the board of trustees, faculty, alumni, and graduates assembled at "The Arena," West Thirty-first Street, where the dinner of the Class of '98 was served, and after the greater portion of the menu was disposed of the following toasts were responded to: "New York College of Veterinary Surgeons," Harry D. Gill, V.S.; "Board of Trustees," R. M. Stiver; "Class of '98," Philip C. Finn, V.S.; "The Graduate," Harry D. Gill, V.S.; "The Alumni," James H. Ferster, V.S.; "Lost Opportunities," J. S. Hopkins, M.D.; "Business Methods," Rush Shippen Huidekoper, M.D., V.S.; "The Profession," Edward N. Leavy, D.V.S.; "The Press," W. Horace Hoskins, D.V.S., and "Farewell Greetings," Arthur Ward Smith, V.S. William H. Wheeler, V.S., was toastmaster. Excellent thoughts and advice came from Professors Gill and Huidekoper as to the thorough work done by the graduates and the hopes indulged in of the work they would be expected to accomplish. Mr. Stiver, of the board of trustees, told of the value of education, of good roads, and how the latter kept pace with advanced education and civilization. The press and its influences, the need and value of higher journalism and its progress were referred to by Dr. Hoskins, and he urged the graduates to early make it a duty to record their experiences and observations and prepare them for publicity and use in the journals. Many excellent thoughts flowed from members of the faculty, alumni, graduates, and the Class of '98, and the hour of midnight quickly rolled round amid the pleasure and enjoyments of the occasion.

